# Genotyping by sequencing reveals contrasting patterns of population structure, ecologically mediated divergence, and long‐distance dispersal in North American palms

**DOI:** 10.1002/ece3.4125

**Published:** 2018-05-08

**Authors:** Anastasia Klimova, Alfredo Ortega‐Rubio, David L. J. Vendrami, Joseph I. Hoffman

**Affiliations:** ^1^ Centro de Investigaciones Biologicas del Noroeste S.C. La Paz Baja California Sur Mexico; ^2^ Department of Animal Behaviour Bielefeld University Bielefeld Germany

**Keywords:** Arecaceae, Baja California peninsula, genotyping by sequencing (GBS), human‐mediated dispersal, single nucleotide polymorphism (SNP)

## Abstract

Comparative studies can provide powerful insights into processes that affect population divergence and thereby help to elucidate the mechanisms by which contemporary populations may respond to environmental change. Furthermore, approaches such as genotyping by sequencing (GBS) provide unprecedented power for resolving genetic differences among species and populations. We therefore used GBS to provide a genomewide perspective on the comparative population structure of two palm genera, *Washingtonia* and *Brahea*, on the Baja California peninsula, a region of high landscape and ecological complexity. First, we used phylogenetic analysis to address taxonomic uncertainties among five currently recognized species. We resolved three main clades, the first corresponding to *W. robusta* and *W. filifera*, the second to *B. brandegeei* and *B. armata*, and the third to *B. edulis* from Guadalupe Island. Focusing on the first two clades, we then delved deeper by investigating the underlying population structure. Striking differences were found, with GBS uncovering four distinct *Washingtonia* populations and identifying a suite of loci associated with temperature, consistent with ecologically mediated divergence. By contrast, individual mountain ranges could be resolved in *Brahea* and few loci were associated with environmental variables, implying a more prominent role of neutral divergence. Finally, evidence was found for long‐distance dispersal events in *Washingtonia* but not *Brahea*, in line with knowledge of the dispersal mechanisms of these palms including the possibility of human‐mediated dispersal. Overall, our study demonstrates the power of GBS together with a comparative approach to elucidate markedly different patterns of genomewide divergence mediated by multiple effectors.

## 1 INTRODUCTION

Comparative phylogeographic studies of codistributed species are of paramount importance in decoding how shared geological and ecological histories may have affected contemporary species and populations (Papadopoulou & Knowles, 2016). By comparing the spatial genetic structure of multiple codistributed species, comparative studies allow the assessment of phylogeographical congruence, which is a baseline for historical inference (Avise, 2000; Garrick, Rowell, Simmons, Hillis, & Sunnucks, 2008; Hickerson et al., 2010; Hoffman, Clarke, Linse, & Peck, 2011). Moreover, a general understanding of how species and populations responded to past challenges might provide us with insights into how present and future human activities will impact the planets biota (Avise, Bowen, & Ayala, 2016).

It is also becoming increasingly recognized that interactions between organisms and their environment can shape the distribution of spatial genetic variation and lead to local adaptation (Anderson, Willis, & Mitchell‐Olds, 2011). This in turn may reduce the amount of gene flow between populations, ultimately putting them on the path to speciation (Rundle & Nosil, 2005; Schluter, 2000). The primary difference between ecologically mediated divergence and neutral divergence is that the former results in a pattern of isolation by environment (IBE) in which genetic and environmental distances are positively correlated independently of geographic distance (Shafer & Wolf, 2013; Wang & Bradburd, 2014). By contrast, the neutral divergence model emphasizes the role of geographic isolation in restricting the exchange of migrants between populations, which results in a pattern of isolation by distance (IBD) that is frequently observed in natural populations (Prunier et al., 2017; Wright, 1943).

Although species that share the same landscape may face similar selective environments, their capacity to adapt to these environments may be species‐ or even population‐specific and can depend on both extrinsic and intrinsic factors, including the strength and nature of selection, the amount of genetic diversity, and the extent of phenotypic plasticity (Sork, Gugger, Chen, & Werth, 2016). Consequently, species inhabiting a common landscape may vary in their capacity to adapt to ecological gradients and changing environments. Assessing adaptive responses among related species within shared heterogeneous landscapes may thus help us to understand patterns of biodiversity. A major step toward this goal is identifying to what extent ecology, gene flow and genomic architecture contribute toward variability in the evolutionary potential of multiple‐related species inhabiting the same landscape (Raeymaekers et al., 2017).

In this regard, the Baja California peninsula, with its heterogeneous array of landscapes and habitats varying from tropical deciduous forests and mesic oases to xeric desert scrub mountains and low altitude arid plains, is of particular interest (Dolby, Bennett, Lira‐Noriega, Wilder, & Munguia‐Vega, 2015). The peninsula was formed around 5–10 million years ago (mya), when tectonic forces gave rise to the Gulf of California and separated a narrow section of land from the Mexican mainland. Currently, a series of mountain ranges, most prominently Sierra San Pedro Martir, Sierra Libertad, and Sierra La Giganta, run in succession along the peninsula from north to south. It has been suggested that the uplift of these major sierras probably began around 6–10 mya and that their formation was tied directly to the same tectonic forces that opened the Gulf of California and created the San Andreas Fault system (Martín‐Barajas, 2014; Mueller, Kier, Rockwell, & Jones, 2009). Together, the sierras constitute a more or less continuous mountainous backbone that separates ecosystems sloping east into the Gulf of California from those running westwards into the Pacific and also creates a complex landscape gradient along the entire peninsular that is reflected in a very high diversity of environments (Riemann & Ezcurra, 2007). This unique natural setup, characterized by unusually high levels of both landscape and ecological complexity, could conceivably shape the population structure of species inhabiting the peninsula via both neutral and ecologically mediated processes, with their relative importance depending on intrinsic qualities of the species in question (Dolby et al., 2015).

An additional dimension is provided by the more recent history of the Baja California peninsula, which experienced a notable ecological shift involving progressive aridification after the Last Glacial Maximum (Lindell, Ngo, & Murphy, 2006; Riddle, Hafner, Alexander, & Jaeger, 2000). Drastic changes in precipitation across the peninsula are believed to have led to several endemic species becoming either locally extinct or restricted to small isolated sites such as oases and canyons (Bernardi, Ruiz‐Campos, & Camarena‐Rosales, 2007; Wehncke, López‐Medellín, & Ezcurra, 2010). This major ecological transition is thought to have most strongly affected species dependent on freshwater such as amphibians (Grismer, 2002), fishes (Bernardi et al., 2007) and many plants (León de la Luz, 2014; Wehncke & López‐Medellín, 2014). However, the sessile nature of plants renders them particularly susceptible to the influence of fine‐scale environmental heterogeneity (Sork et al., 2016), which makes the flora of the Baja peninsula ideally suited to exploring the relative contributions of geography and ecology toward species‐specific responses to aridification.

Palms (Arecaceae) are a species‐rich taxonomic group that has long been considered a model plant family for evolutionary and ecological studies in the tropics (Bacon et al., 2016; Couvreur & Baker, 2013; Kissling et al., 2012; Savolainen et al., 2006). Although palms mainly occur in tropical and subtropical climates (Tregear, Rival, & Pintaud, 2011), a few species can also be found in desert regions (Dransfield et al., 2008; Tomlinson, 2006). Palm populations on the Baja California peninsula are widely considered to be relicts of historically more widespread and continuous populations that are now largely confined to sites where permanent water exists either above or below the ground (Axelrod, 1958; Bacon, Baker, & Simmons, 2012; Cornett, 1985a,b; Cornett, Glen, & Stewart, 1986; Felger & Joyal, 1999; Grismer & McGuire, 1993). This is reflected by the fossil record, which shows that during the late Cretaceous, palms were common across North America and extended much further than their current geographic distribution (Couvreur, Forest, & Baker, 2011; Harley, 2006). Today, the only suitable natural habitats for tropical species like palms on the Baja California peninsula can be found in small isolated pockets at the bottoms of canyons or oases that are separated by a continuous expanse of mountainous deserts and xeric shrubland (Grismer, 2000; Hafner & Riddle, 2011; Minnich, Franco‐Vizcaíno, & Salazar‐Ceseña, 2011; Wehncke & López‐Medellín, 2014).

On the Baja peninsula, the Arecaceae is represented by two native North American palm genera, *Washingtonia* and *Brahea* (Garcillán, Vega, & Martorell, 2012; Minnich et al., 2011)*. Washingtonia* is represented by two species, *W. robusta* and *W. filifera*, although in general taxonomic relationships within this genus remain poorly resolved (Felger & Broyles, 2007; Felger & Joyal, 1999; Henderson, Galeano, & Bernal, 1995; McClintock, 1978) as they are mainly based on morphological characters such as size, leaf shape and inflorescence structure. *W. robusta* is more abundant on the southern part of the peninsula, while *W. filifera* mainly occupies the northern peninsula as well as southeastern California (Minnich et al., 2011). *W. robusta* is also found in a few riparian canyons at the southern edge of the Sonoran Desert on the Mexican mainland (Felger & Joyal, 1999).

The *Brahea* complex comprises nine species, two of which are endemic and restricted to the Baja California peninsula (*B. brandegeei* and *B. armata*) and one (*B. edulis*) to Guadalupe Island, which lies 260 km off the Pacific coast of the peninsula (Garcillán et al., 2012; Moran, 1996; Oberbauer, 2005). *Brahea* is arguably the least studied genus of American palms, and consequently, relationships between and within *Brahea* species have not been clearly described (Henderson et al., 1995; Minnich et al., 2011; Quero, 1992). Nevertheless, *B. brandegeei* has been described as occupying the southern half of the Baja California peninsula, from Sierra La Laguna at the southernmost tip of the peninsula to Sierra San Francisco in the northern Baja California Sur (Felger, Johnson, & Wilson, 2001; Minnich et al., 2011), while *B. armata* has been described as extending northwards from the state line of Baja California Sur in the central peninsula to just south of the United States–Mexico border (Franco‐Vizcaíno, López‐Beltrán, & Salazar‐Ceseña, 2007; Wiggins, 1980). However, the exact distributional limits of *B. brandegeei* and *B. armata* remain somewhat unclear due to taxonomic uncertainties (Felger & Joyal, 1999; Felger et al., 2001; Henderson et al., 1995). Finally, *B. edulis* is an endemic species found uniquely on Guadalupe Island (29°N, 118°W), a seven‐million‐year‐old volcanic island located 260 km west of the Baja California peninsula (Batiza, 1977). It is generally believed that Guadalupe Island has never been in contact with either the Mexican mainland or the Baja peninsula, which would imply that *Brahea* palms colonized the island via long‐distance dispersal. Currently, these palms mainly occupy a small area of fog oasis in the far northern part of the Island (Garcillán et al., 2012; León de la Luz, Rebman, & Oberbauer, 2003; Oberbauer, 2005).

From an ecological perspective, palm populations of the Baja peninsula generally form small local colonies or elongate galleries, with plants growing almost entirely in areas with supplemental water, that is, in close vicinity to springs or along water courses (Franco‐Vizcaíno et al., 2007; Minnich et al., 2011). *Washingtonia* and *Brahea* co‐occur along the slopes of most of the sierras, including Sierra Juarez, Sierra Asamblea, Sierra Mechudo and Sierra La Laguna (Klimova, Hoffman, Gutierrez‐Rivera, Leon de la Luz, & Ortega‐Rubio, 2017; Minnich et al., 2011). However, they differ somewhat in their ecological requirements, with their estimated niche overlap ranging from around 0.5–0.7 depending on the statistic used (Klimova et al., 2017). In particular, *Washingtonia* palms have stricter ecological requirements than *Brahea* palms and therefore occupy a smaller total area of the Baja California peninsula (Minnich et al., 2011). They are most commonly found at low‐elevation oases where water is relatively plentiful and temperatures tend to be warmer and more stable. By contrast, *Brahea* palms are capable of tolerating drier conditions and thus occupy a much wider elevational range. Accordingly, *W. filifera* and *W. robusta* are seldom found above 1,000 m, whereas *B. armata* may grow at elevations as high as 1,400 m in the Sierra San Pedro Martir and *B. brandegeei* occurs at up to around 1,700 m in the Sierra La Laguna (Minnich et al., 2011).

Species‐specific modes and patterns of dispersal are another key determinant of genetic structure in the Arecaceae (Eiserhardt, Svenning, Kissling, & Balslev, 2011). Although vertebrates such as birds, bats, coyotes, and foxes have been proposed as possible seed dispersal agents for both *Washingtonia* and *Brahea*, the effect of these agents on the population structure and genetic diversity of palms has not been evaluated (Cornett, 2008; Silverstein, 2005). Furthermore, recent studies of *Brahea* palms suggest that water pulses may be far more significant in terms of seed dispersal than birds and small mammals (Wehncke, López‐Medellín, & Ezcurra, 2009; Wehncke et al., 2010). This leads to the prediction that dispersal in *Brahea* palms should occur mainly within watercourses or canyons and hence that connectivity among sierras will be restricted. A further complication is the potential influence of humans, which is predicted to be stronger for *Washingtonia* as this genus is widely considered to have been favored by indigenous people over *Brahea* as an important source of both food and building materials (Felger & Joyal, 1999; Felger & Moser, 1985; Minnich et al., 2011).

For the reasons described above, *Washingtonia* and *Brahea* palms provide an attractive system for investigating the contributions of neutral, non‐neutral and human‐mediated effects toward population structure in a comparative context. However, despite the emblematic status of these desert palms, many aspects of their taxonomy remain ambiguous (Minnich et al., 2011). This is partly because previous genetic studies do not always support currently recognized species. For example, Bacon et al. (2012) did not find any evidence in support of the designation of *W. robusta* and *W. filifera* as separate species based on three plastid and three nuclear genes. This ambiguity is to some extent reflected by a more recent study of *Washingtonia* and *Brahea* palms from the Baja California peninsula and adjacent areas, again based on nuclear and chloroplast sequence data (Klimova et al., 2017). Here, both genera were found to exhibit low genetic diversity and minimal structuring within the peninsula, similarly to previously reported for *W. filifera* in a small region of the Californian mainland using allozymes (McClenaghan & Beauchamp, 1986). However, *W. filifera* could be distinguished from *W. robusta* based on chloroplast but not nuclear DNA, whereas *B. edulis* was divergent from its peninsular sister species based on nuclear, but not chloroplast DNA. Such incongruences together with the generally low genetic resolution provided by the markers used in this study precluded comprehensive hypothesis testing, and thus, more detailed inferences could not be made. Consequently, at the present time, even the geographic limits of the *Washingtonia* and *Brahea* species present on the Baja California peninsula remain unclear (Henderson et al., 1995; Klimova et al., 2017; Minnich et al., 2011), while virtually nothing is known about relationships among populations from different sierras.

Studies based on one or handful of genes like those described above may also suffer from a number of biases related to stochastic processes (Heath, Hedtke, & Hillis, 2008; Moore, 1995; Rokas & Carroll, 2005). However, approaches capable of genotyping thousands of single nucleotide polymorphisms (SNPs) such as genotyping by sequencing (GBS) are capable of providing much greater coverage of the genome (De Donato, Peters, Mitchell, Hussain, & Imumorin, 2013; Elshire et al., 2011). Recent simulation and empirical studies suggest that these approaches should be superior to a handful of markers at capturing variation in drift, selection, recombination, and mutation (Morin, Luikart, & Wayne, 2004) and thereby provide a more accurate depiction of population differentiation (Spinks, Thomson, & Shaffer, 2014; Vendrami et al., 2017) and genetic diversity (Fischer et al., 2017; Hoffman et al., 2014).

To address the issues described above, we combined GBS with near‐exhaustive sampling of all five native *Washingtonia* and *Brahea* palm species present on the Baja California peninsula and Guadalupe Island. The resulting data were then analyzed on two levels. First, we attempted to resolve taxonomic relationships and from there to delimit the geographic boundaries of each species on the peninsula. Second, we focused within the major clades identified by the former analysis and conducted population genetic analyses to uncover patterns of population structure on the Baja peninsula and investigate the potential underlying drivers. Our main working hypotheses were as follows: (1) We expected to find genomic support for most if not all of the currently recognized species, with the possible exception of *W. robusta* and *W. filifera*; (2) as water pulses appear to be an important mediator of dispersal in *Brahea* palms, we hypothesized for this genus that dispersal would be mainly restricted within watersheds, which should be reflected in stronger population structure than in *Washingtonia* and potentially manifested in a pattern whereby each sierra is genetically distinct; (3) we hypothesized that ecologically mediated selection should be comparably more important in *Washingtonia* palms due to their stricter ecological requirements, which could potentially lead to an IBE pattern. By contrast, we expected *Brahea* palms to be more influenced by neutral processes and thus to exhibit an IBD pattern; (4) finally, due to the long historical association between *Washingtonia* palms and humans, we hypothesized that population structure in *Washingtonia* could potentially also have been influenced by human‐mediated translocation events.

## 2 MATERIALS AND METHODS

### 2.1 Sample collection

**Figure 1:**
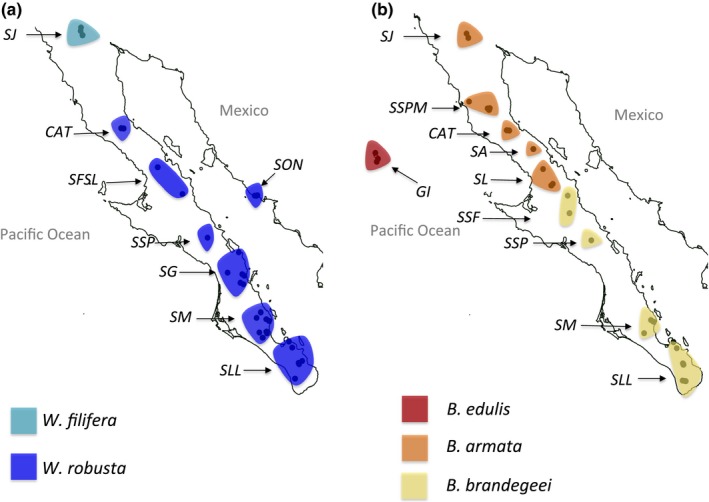
Maps showing the oases (points) and the sierras (shape files) from which (a) *Washingtonia* and (b) *Brahea* palms were sampled. The full names of the oases and sample sizes are given in Table S1. The colors of the shapefiles correspond to species (as defined in Minnich et al., 2011): (a) *W. robusta* on the Baja peninsula and Mexican mainland and *W. filifera* at *SJ*; (b) *B. edulis* at the Guadalupe Island (*GI*), *B. armata* at *SL, SA, CAT, SSPM,* and *SJ* and *B. brandegeei* at *SLL, SM, SPP,* and *SSF*

We collected a total of 190 leaf samples from all five palm species native to the Baja California peninsula and Guadalupe Island (Figure 1; Table S1). Our sample size reflects the difficulty of collecting samples from endemics that are locally rare and can only be found at small and isolated stands that can often only be reached by foot. We collected specimens from virtually all accessible oases during two consecutive field seasons, each lasting ~4 weeks. Whenever possible, we avoided sampling immediately adjacent individuals as Migliore et al. (2013) showed that around a third of adjacent sampled individuals of a relict shrub species were clones. For comparison, we also included two populations of *W. robusta* from Sonora on the Mexican mainland. We were therefore able to cover the full distributional ranges of three *Brahea* species (*B. edulis, B. armata,* and *B. brandegeei*) and one *Washingtonia* species (*W. robusta*), while *W. filifera* could only be sampled from its southern distributional limit in Sierra Juarez, Mexico (Figure 1; Table S1). Specimens were assigned to taxa based on species distributions given by Minnich et al. (2011). Within species, populations were defined based on the sierra from which the samples were collected. For *Washingtonia*, we specified eight populations corresponding to palms from (1) Sierra La Laguna (*SLL*), (2) Sierra Mechudo (*SM*), (3) Sierra Giganta (*SG*), (4) Sierra San Pedro (*SSP*), (5) Sierra San Francisco combined with Sierra Libertad (*SFSL*), (6) Cataviña (*CAT*), (7) Sierra Juarez (*SJ*), and (8) Mexican mainland, state of Sonora (*SON*). For *Brahea*, we specified nine populations on the Baja peninsula corresponding to (1) Sierra La Laguna (*SLL*), (2) Sierra Mechudo (*SM*), (3) Sierra San Pedro (*SSP*), (4) Sierra San Francisco (*SSF*), (5) Sierra Libertad (*SL*), (6) Sierra Asamblea (*SA*), (7) Cataviña (*CAT*), (8) Sierra San Pedro Martir (*SSPM*), and (9) Sierra Juarez (*SJ*) (Figure 1; Table S1).

### 2.2 Genetic analysis

Total genomic DNA was extracted from silica‐dried leaves using a modified CTAB protocol (Gutierrez‐Rivera in preparation), and 50 μl of DNA from each sample was sent to the Cornell Institute of Genomic Diversity for library preparation and GBS (Elshire et al., 2011). Each DNA extract was digested using the restriction enzyme PstI, and subsequently, a sample‐specific barcoded adapter and a common adapter were ligated to the sticky ends of fragments to allow for sample discrimination after pooling. A unique barcoded adapter was used for each sample plus two negative controls, giving a total of 192 barcodes employed. Next, samples from the two different genera were pooled together into two separate libraries that were each 100 bp single‐end sequenced on one lane of an Illumina HiSeq 2000.

The resulting raw reads were processed using the TASSEL 3.0 pipeline (Bradbury et al., 2007) which implements the customized workflow specifically designed for GBS data described by Glaubitz et al. (2014). Specifically, all identical reads were first collapsed into tags and the number of reads used for the generation of each tag was reported. Before this step, in order to ensure the usage of exclusively high‐quality reads, only reads containing a barcode, the correct restriction enzyme cutting site and with no Ns were retained. After removing the barcode sequences, the remaining reads were then trimmed to a final length of 64 bp, with any reads containing a second restriction site being truncated. Then, a “master” list of tags was created containing only tags built from at least three reads (i.e., the minimum depth of coverage of a tag had to be three reads). This number was chosen because lower values will result in more sequencing errors being included, while higher values will result in the exclusion of rare alleles. We also chose a value of three because this allowed tags containing up to three sequencing errors to be assembled, which increased the depth of coverage of non‐error positions. Sequencing errors were subsequently removed by filtering out SNPs with low minor allele frequencies (MAF) as described below.

Finally, the master list of tags was aligned to the date palm (*Phoenix dactylifera*) reference genome (date palm, downloaded from https://www.ncbi.nlm.nih.gov/genome/ in June 2016) using the software BWA (Li & Durbin, 2009) to produce a SAM file. After conversion of this file with SAMConvertor, the tbt2vcf plug‐in within TASSEL 3.0 was used to call SNPs from tags that aligned to unique locations in the reference genome, which were then exported in VCF format. SNPs with MAF below than 0.01 were then removed from the dataset in order to filter out false SNPs originating from sequencing errors while retaining genuine variants including moderately rare alleles. InDels were then removed from the variant dataset, which was further filtered to discard chloroplast and mitochondrial variants, SNPs that were not bi‐allelic and loci carrying only heterozygote genotypes, which represent putatively paralogous loci. Next, in order to avoid linkage between loci, we pruned the SNPs for linkage disequilibrium using the thinning option in VCFTOOLS (Danecek et al., 2011) with a 5 k sliding window. Finally, we removed loci with more than 20% missing genotypes. This conservative measure was taken to minimize the frequency of gaps and thereby to maximize the robustness of our downstream analyses. The final dataset was used for phylogenetic analysis and to compare levels of genetic diversity between the two genera. After that, we generated two separate datasets for *Washingtonia* and *Brahea,* respectively, to allow finer‐scale population genetic analyses. These datasets were generated for each genus using the same filtering steps described above. Additionally, five individuals with more than 30% missing data (four *Washingtonia* and one *Brahea*) were excluded from further analyses. Manipulations of the VCF files were carried out using VCFTOOLS. Inbreeding coefficients and observed and expected heterozygosities were calculated for each palm genus separately using PLINK 1.9 (Chang et al., 2015).

### 2.3 Comparative phylogenetic analyses

In order to resolve taxonomic relationships among the morphologically defined species and to delimit the geographic boundaries of each species, we performed Bayesian phylogenetic reconstruction using the SNAPP package (Bryant, Bouckaert, Felsenstein, Rosenberg, & Choudhury, 2012), within the program BEAST2 (Bouckaert et al., 2014). Due to computational limitations, we were unable to analyze the full dataset and therefore restricted our analysis to a random selection of three individuals per population, resulting in a total of 54 individuals representing two palm genera. We used the default prior and model parameters, including the defaults for u and v (the backward and forward mutation rates, respectively) and ran a single Markov chain Monte Carlo (MCMC) chain of 2,000,000 iterations with sampling every 1,000 steps. After running the full dataset, we then analyzed each genus separately using same parameters as before. Acceptable mixing (requiring effective sample size values to be at least 200) and convergence were checked by visual inspection of the posterior samples using TRACER (Rambaut, Suchard, Xie, & Drummond, 2014). We used a burn‐in of 10% and visualized the distribution of trees using DENSITREE 2.1 (Bouckaert, 2010).

### 2.4 Population structure analyses

Based on the results of phylogenetic analyses (see Section 2.1), we decided to exclude *B. edulis* from population‐level analyses or where appropriate to use it as an outgroup. Downstream population genetic analyses were conducted after defining samples from each of the sierras as *a priori* populations (Figure 1; Table S1). First, pairwise *F*_st_ values among sierras were calculated within the program GENODIVE (Meirmans & Van Tienderen, 2004) with statistical significance determined on the basis of 10,000 permutations. Then, we used sNMF 1.2 (Frichot, Mathieu, Trouillon, Bouchard, & François, 2014) to estimate individual admixture coefficients and to determine the most probable number of genetic clusters (*k*) present within each genus. sNMF was chosen in preference to more computer‐intensive approaches such as STRUCTURE (Pritchard, Stephens, & Donnelly, 2000) as it uses fast and efficient sparse non‐negative matrix factorization algorithms that considerably reduce the computational burden without any appreciable loss of accuracy (Frichot et al., 2014; Popescu, Harper, Trick, Bancroft, & Huber, 2014; Wollstein & Lao, 2015). The best *k* value was inferred by calculating cross‐entropy values from multiple runs with *k* set between one and ten. Robustness of the results was assessed by running five replicates for the best value of *k* using an alpha regularization parameter of 100. We conducted model averaging of individual ancestry coefficients across replicates and calculated the average pairwise similarity of individual assignments across runs using CLUMPP (Jakobsson & Rosenberg, 2007). Finally, we converted the SNP data into a matrix of individual pairwise genetic distances using the R package STAMPP (Pembleton, Cogan, & Forster, 2013) and generated a phylogenetic network using the NEIGHBORNET algorithm (Bryant & Moulton, 2004) within SPLITSTREE 4.14.4 (Huson & Bryant, 2006).

### 2.5 Population splits and migration modeling

We used the approach of Pickrell and Pritchard (2012) to infer the population history of the palm taxa using genome‐wide allele frequency data as implemented in TREEMIX 1.12 (Pickrell & Pritchard, 2012). TREEMIX infers gene flow between populations by simultaneously analyzing population divergence and admixture. On the resulting maximum‐likelihood (ML) tree, migration events are represented by edges that connect populations via admixture. SNP data for *Washingtonia* and *Brahea* were converted from a diploid genotype format into population‐level allele counts using the python script plink2treemix.py (available with TREEMIX). Each population was represented by individuals from a given sierra as described previously. For *Washingtonia*, we used the most divergent sierra (SJ) as an outgroup, while for *Brahea*, we used *B. edulis* as an outgroup. We first generated a maximum‐likelihood graph with no migration events based on 1,000 bootstrap replicates. Then, we tested for between one and ten migration events per taxon (m1–m10) and performed likelihood ratio tests to allow stepwise comparison of log‐likelihood values between each pair of migration events. After that we formally tested for admixture using the “three‐population test” (Patterson et al., 2012) implemented in TREEMIX. This is a formal test that can provide evidence of admixture even in the presence of past migration events (Patterson et al., 2012). It allows detection of the presence of admixture in population X from other two populations, A and B; if the value of f3 (X; A, B) is negative, then the deviation from “treeness” is detected and X appears to be a mixture of A and B.

### 2.6 Isolation by distance and ecological divergence

Isolation by distance (IBD) and dispersal barriers are known to contribute toward the geographic structuring of genetic variation in many organisms. We therefore used the Isolation By Distance Web Service 3.23 (Jensen, Bohona, & Kelley, 2005) to perform reduced major axis regression and Mantel tests based on 10,000 randomizations of the datasets. Geographic great‐circle distance and pairwise genetic distances between individuals were calculated using the Geographic Distance Matrix Generator 1.2.3 (Ersts, 2016) and the R package STAMPP (Pembleton et al., 2013) respectively. Local adaptation can also be manifested in correlations between genetic and environmental distances (Frankham, Ballou, & Briscoe, 2002). We therefore used Mantel and partial Mantel tests as implemented in the R package VEGAN 2.4‐0 (Oksanen et al., 2013) to test for correlations between genetic and environmental distances, the latter being generated using the “dist” function in R. As *Washingtonia* and *Brahea* are affiliated to humid tropical climates and are frost sensitive, we expected climatic variables such as the mean temperature of the coldest quarter, precipitation of the driest quarter, and the aridity index to have the greatest influence on these genera. The above‐mentioned ecological information was therefore downloaded from WorldClim with the resolution of 30 arc‐seconds (~1 km) (Hijmans, Cameron, Parra, Jones, & Jarvis, 2005) and from the Global Aridity and PET database (Zomer, Trabucco, Bossio, & Verchot, 2008) as a set of raster layers. Mantel tests were then performed between each genetic and environmental distance matrix, and these analyses were also repeated as partial Mantel tests controlling for geographic distance. Statistical significance was determined using Pearson tests based on 10,000 permutations of the data.

### 2.7 Detection of outlier loci associated with environmental variables

Environmental variables showing significant associations with genetic distance in the above analyses (specifically, mean temperature of the coldest quarter in *Washingtonia* and precipitation in the driest quarter for *Brahea*, see Section 2.1) were further investigated by testing for signatures of local adaptation using the R package LEA (Landscape Genomics and Ecological Association Test, Frichot, Schoville, Bouchard, & François, 2013; Frichot & François, 2015) and SAMβADA (Stucki et al., 2016). The first of these programs uses latent factor mixed models LFMMs to detect loci exhibiting unusual associations with environmental variables compared to the genomic background. We chose to use this program as it can account for the underlying population structure by introducing “latent factors” while simultaneously estimating random effects due to population history and isolation by distance. We ran 10,000 iterations of the Gibbs sampling algorithm with the first 5,000 iterations discarded as burn‐in. *Z* scores from five independent replicate runs were then combined and the resulting *p*‐values were adjusted for the false discovery rate (FDR) as described in Benjamini and Hochberg (1995) with an alpha level of 0.05.

The second package, SAMβADA, implements logistic regressions to model the probability of observing a particular genotype at each marker given the environmental conditions at the sampling locations (Joost et al., 2007). We chose the multivariate option as this allows a combination of predictor variables to be simultaneously assessed, thereby reducing the occurrence of spurious genotype by environment associations (Stucki et al., 2016). The two predictor variables for both *Washingtonia* and *Brahea* were the genetic groups identified with SPLITSTREE and the respective ecological variable identified using partial Mantel tests. Any SNPs associated with the main genetic groups were then discarded and only those SNPs associated with ecological variables were retained. Statistical significance was determined using both log‐likelihood ratio and Wald tests (Joost et al., 2007) and FDR was applied to the resulting *p*‐values with an alpha level of .05.

## 3 RESULTS

### 3.1 Genomic data

We subjected 190 palm samples to GBS, generating a total of 296,358,035 high‐quality barcoded reads, which were assembled into 549,976 tags that aligned uniquely to the *P. dactylifera* reference genome. From these data, we called a total of 26,565 SNPs. After quality filtering, removing InDels, retaining only bi‐allelic nuclear SNPs, LD filtering and removing loci with more than 20% missing data, this was reduced to 2,063 SNPs distributed over 724 scaffolds (median = one SNP per scaffold, range = 1–30, see Figure S1). The final dataset comprised 514 diagnostic SNPs (i.e., loci that were fixed for different alleles in the two genera) plus 1,549 polymorphic SNPs, of which 183 were polymorphic in both genera, 312 were polymorphic only in *Washingtonia* and 1,054 were polymorphic only in *Brahea* (Figure S2). Additionally, we generated separate datasets for each palm genus comprising 85 *Washingtonia* palms genotyped at 1,462 polymorphic SNPs and 79 *Brahea* palms (excluding *B. edulis*) genotyped at 2,050 polymorphic SNPs. Observed heterozygosity was lower in *Washingtonia* (0.098, 97.5% CI = 0.089–0.108) than in *Brahea* (0.139, 97.5% CI = 0.131–0.146), while the opposite was found for expected heterozygosity (*Washingtonia*: 0.209, 97.5% CI = 0.200–0.217; *Brahea*: 0.175, 97.5% CI = 0.168–0.182). Consistent with differences in observed heterozygosity, the genomic inbreeding coefficient *Fhat3* was higher in *Washingtonia* (0.47, CI: 0.37–0.57) than in *Brahea* (0.25, CI: 0.21–0.29).

### 3.2 Phylogenetic relationships

**Figure 2:**
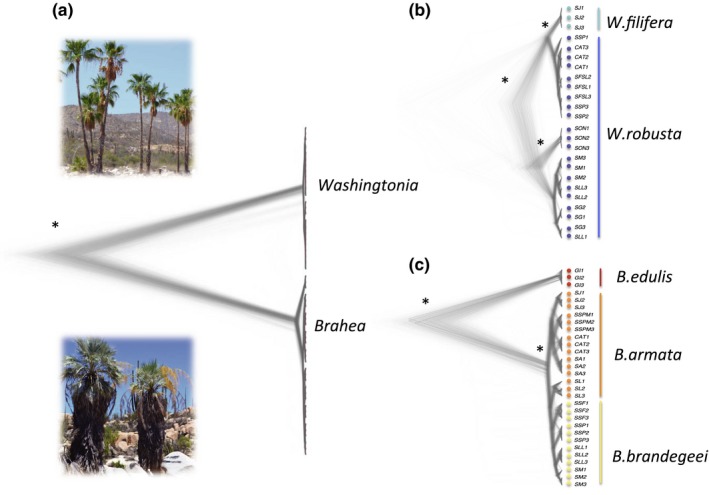
Bayesian reconstruction of the phylogenetic relationships (a) between *Washingtonia* and *Brahea*; (b) within *Washingtonia*; (c) within *Brahea*. Colored lines delimit morphologically defined species according to Minich et al. (2011), and colored dots correspond to the sampled populations. Major nodes with high posterior probability support (*>*0.95) are indicated in asterisks (*)

To elucidate taxonomic relationships, we constructed a Bayesian phylogenetic tree based on a subset of 54 *Washingtonia* and *Brahea* individuals (Figure 2). As expected, the two genera were clearly resolved as distinct and deeply divergent monophyletic clades (Figure 2a). However, when each genus was analyzed separately, we found little evidence in support of the majority of morphologically defined species. Specifically, *W. filifera* did not form a monophyletic group, but instead grouped together with *W. robusta* palms from the northern Baja California peninsula (*SSP*,* SFSL*, and *CAT*) as shown in Figure 2b. Furthermore, greater divergence was observed between *W. robusta* from the Mexican mainland (*SON*) and its peninsular conspecifics than between *W. filifera* and *W. robusta* from the northern Baja California peninsula. This suggests that any genetic differences between *W. filifera* and *W robusta* are smaller than the magnitude of intraspecific variation within *W. robusta*.

By contrast, two distinct monophyletic clades were resolved in *Brahea*, the first corresponding to *B. edulis* from Guadalupe Island and the second comprising *B. armata* and *B. brandegeei* (Figure 2c). Within the peninsular clade, individuals diverged from each other not based on morphological species designations but according to the sierras they were collected from. On the bases of the above analyses, we therefore defined three major palm clades on the Baja California peninsula and Guadalupe Island comprising (1) *W. robusta* and *W. filifera*; (2) *B. brandegeei* and *B. armata*; and (3) *B. edulis*.

### 3.3 Population structure

Next, we carried out population genetic analyses to investigate the comparative population structure of *Washingtonia* and *Brahea* palms on the Baja California peninsula. These analyses were conducted separately for the two clades identified above corresponding to *W. robusta* and *W. filifera* (forthwith referred to as *Washingtonia*) and *B. brandegeei* and *B. armata* (forthwith referred to as *Brahea*). Strong population structure was found in both genera, with majority of pairwise comparisons among sierras yielding moderately large and highly significant *F*_st_ values (Tables S2 and S3). For *Washingtonia*, the greatest genetic differences were observed between the peninsular and mainland localities (*F*_st_ = 0.55–0.86, *p *<* *.001) as well as between the northernmost population of *SJ* and the other sierras (*F*_st_ = 0.46–0.86, *p *<* *.001). Additionally, sierras of the northern (*CAT*,* SFSL*, and *SSP*) and southern (*SG*,* SM,* and *SLL*) regions of the Baja peninsula were significantly differentiated from one another (*F*_st_ = 0.27–0.56, *p *<* *.01), whereas negligible structure was found within each of these regions (Table S2). By contrast, in *Brahea*, all pairwise *F*_st_ comparisons among sierras within the peninsula were statistically significant (Table S3).

**Figure 3:**
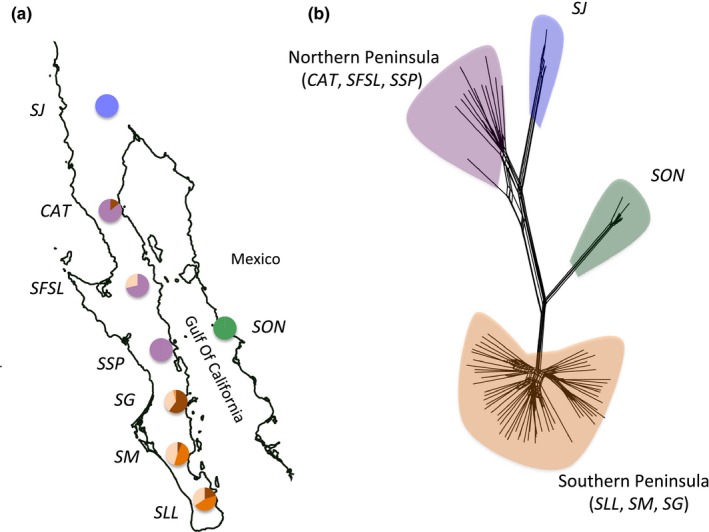
Population structure of *Washingtonia* palms. Panel (a) shows the results of cluster analysis within sNMF, with pie charts indicating the geographic distribution of six inferred genetic clusters (each coded by a different color). Panel (b) shows a phylogenetic network generated by SPLITSTREE

**Figure 4:**
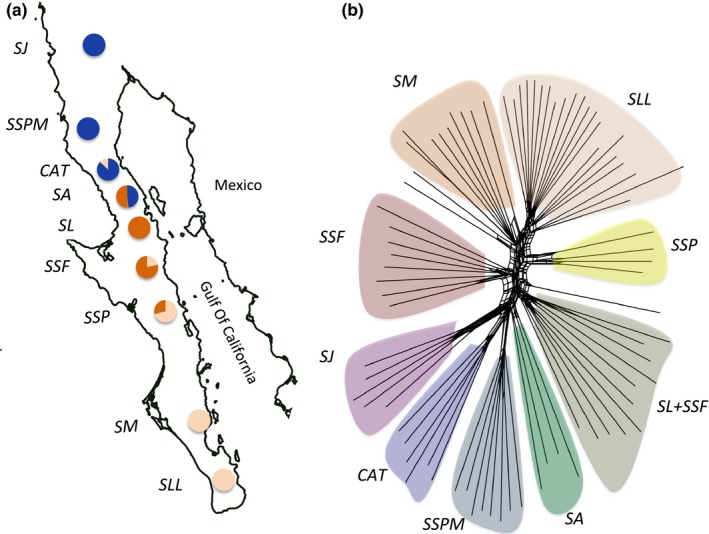
Population structure of *Brahea* palms. Panel (a) shows the results of cluster analysis within sNMF, with pie charts indicating the geographic distribution of three inferred genetic clusters (each coded by a different color). Panel (b) shows a phylogenetic network generated by SPLITSTREE

To uncover the main genetic clusters present within *Washingtonia* and *Brahea*, we used admixture estimation and individual clustering within sNMF as well as phylogenetic network inference within SPLITSTREE (see Section 2 for details). Both of these approaches resolved clear groupings and there was general agreement between them on the strength and pattern of population structure. In the case of *Washingtonia*, four main distinct genetic clusters were recovered (Figure 3; Figure S3) corresponding to: (1) the Mexican mainland (*SON*), (2) the southern Baja peninsula (*SLL*,* SM,* and *SG*), (3) the northern Baja peninsula (*SSP*,* SFSL,* and *CAT*), and (4) *Washingtonia* from Sierra Juarez (*SJ*). For *Brahea*, there were some differences in the results depending on the analytical approach used. Specifically, sNMF uncovered three distinct genetic clusters (Figure 4a) representing (1) the southern Baja peninsula (*SLL*,* SM,* and *SSP*), (2) the central Baja peninsula (*SSF*,* SLI,* and *SA*), and (3) the northern Baja peninsula (*CAT*,* SSPM,* and *SJ*). By contrast, the SPLITSTREE analysis (Figure 4b) was more sensitive to the phylogenetic structuring of the peninsular *Brahea* palms and clearly partitioned the samples into nine genetic clusters, each corresponding to a different sierra. The only exceptions were two samples from *SSF* that clustered together with palms from *SL*, two individuals that were misplaced between *SSF* and *SM,* and one individual between *SSP* and *SL*. Intriguingly, individual cluster membership plots (Figure S3) also highlighted the presence of individual palms sampled from the northern sierras that exhibited clear genetic ancestry in the southern sierras. Specifically, four *Washingtonia* palms sampled from two of the northern sierras (*CAT* and *SFSL*) had genotypes indicative of ancestry in the southern sierras *SLL*,* SM,* and *SG*, whereas two *Brahea* individuals sampled from *CAT* had genetic ancestry consistent with *SM* and *SLL*.

### 3.4 Population splits and migration modeling

**Figure 5:**
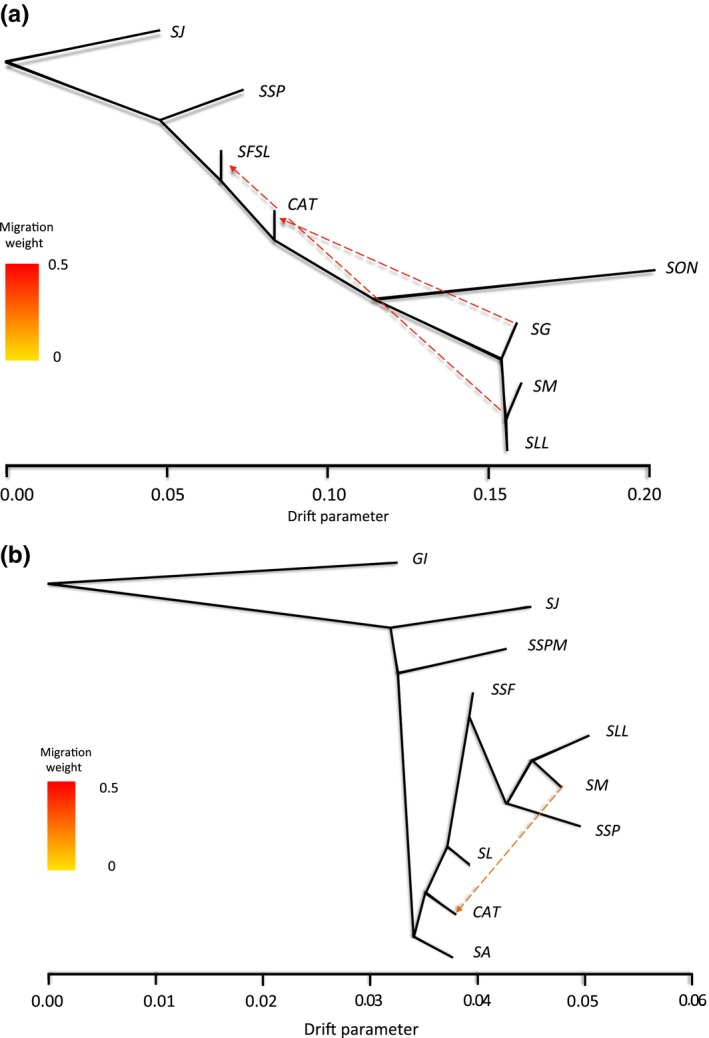
Maximum‐likelihood trees depicting patterns of genetic divergence among (a) *Washingtonia* palms grouped into eight populations and (b) *Brahea* palms grouped into ten populations (see Section 2 for details). Inferred migration events are indicated by dashed lines, with the direction of gene flow indicated by arrows and color intensity reflecting the intensity of gene flow

To investigate the potential cause of some individuals being misassigned to their populations of origin, we modeled population divergence with migration within TREEMIX. The resulting maximum‐likelihood (ML) tree for *Washingtonia* was concordant with the previous results, revealing deep divergence between the mainland and peninsula populations and partitioning of the latter into southern (*SLL*,* SM,* and *SG*) and northern (*SSP*,* SFSL*,* CAT*) groups (Figure 5a). After sequentially testing for between one and ten discrete migration events, we found that the increase in likelihood beyond two migration events was close to zero (Figure S4) and stepwise comparisons of log‐likelihood values lost significance between two and three events (likelihood ratio test, *p *>* *.05). This indicates that the most likely number of migration events among the sierras was two. Exploring this scenario further, we found evidence for unidirectional long‐distance migration spanning around 450 km, from *SG* into *CAT* and from *SM* into *SFSL* (Figure 5a). These migration events were strongly supported by three‐population tests (Table S4).

For the *Brahea* palms, TREEMIX analysis supported the partitioning of the peninsular samples into two major groups comprising the northernmost sierras (*SJ* and *SSPM*) and the rest of the peninsula (Figure 5b). The latter in turn was partitioned into the northern sierras (*SL*,* SA*, and *CAT*) and the southern and mid‐peninsula sierras (*SLL*,* SM*,* SSP*, and *SSF*). Inferred migration events for *Brahea* pointed toward the possible migration of palms between the southern sierra of *SM* and the northern sierra of *CAT* (Figure 5b). However, the overall pattern of increasing log likelihood with the number of migration events was less pronounced than in *Washingtonia* (Figure S4) and none of the migration events were supported by three‐population tests (*Z* scores < −1.96).

### 3.5 Isolation by distance and ecological divergence

Mantel tests revealed strong positive correlations between genetic and geographic distance for both *Washingtonia* and *Brahea* (Mantel's *r* = .685, *p *<* *.0001 and *r* = .609, *p *<* *.0001 respectively, Table 1). Furthermore, Partial Mantel tests revealed significant associations between genetic distance and environmental variables after controlling for geographic distance. Specifically, the mean temperature of the coldest quarter correlated significantly with genetic distance in *Washingtonia* (Partial Mantel test, *r* = .267, *p *<* *.0001), while the amount of precipitation in the driest quarter correlated significantly with genetic distance in *Brahea* (Partial Mantel test, *r* = .09, *p* = .019).

**Table 1:** Mantel and partial Mantel tests summarizing relationships (*r* and associated *p* values) between genetic distance, geographic distance, and climate variables in *Washingtonia* and *Brahea P*‐values * *P* < 0.05; ** *P* < 0.01; *** *P* < 0.001

	*Washingtonia*		*Brahea*	
	Mantel	Partial Mantel	Mantel	Partial Mantel
Geographic distance	0.685***	NA	0.609***	NA
Aridity	−0.059	−0.180	0.068*	−0.230
Mean temperature of the coldest quarter	0.620***	0.267***	0.248***	−0.160
Precipitation of the driest quarter	0.177***	0.010	0.127**	0.09*

### 3.6 Detection of outlier loci associated with environmental variables

In order to investigate the genomic basis of the associations described above, we used two complimentary approaches to test for signatures of local adaptation in *Washingtonia* and *Brahea*. First, latent factor mixed models LFMMs were used to detect loci exhibiting unusual associations with mean temperature of the coldest quarter in *Washingtonia* and precipitation of the driest quarter in *Brahea*. This resulted in the identification of 80 SNPs in *Washingtonia* and 51 SNPs in *Brahea* after table‐wide correction of the corresponding *p*‐values for the false discovery rate. Second, we used a multivariate approach implemented in SAMβADA to test for genotype by environment associations. This approach identified 18 significant associations in *Washingtonia* and none in *Brahea*.

**Figure 6:**
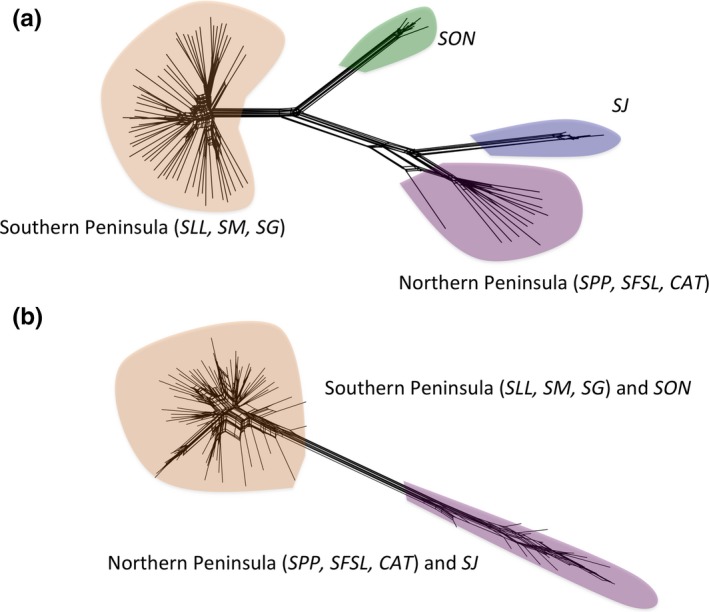
Phylogenetic networks constructed separately for *Washingtonia* using (a) neutral loci; (b) outlier loci

Finally, we asked whether the outlier loci identified by LFMM and SAMβADA (total *n* = 97 for *Washingtonia* and 51 for *Brahea*) resolve contrasting phylogenies to the neutral loci (defined as those loci that were not identified by either program, *n* = 1,365 for *Washingtonia* and 1,999 for *Brahea*). Analysis within SPLITSTREE revealed a striking pattern for *Washingtonia* in which the neutral loci resolved four groups corresponding to the southern Baja peninsula (*SLL*,* SM*, and *SG*), the northern Baja peninsula (*SSP*,* SFSL*, and *CAT*), *SJ* and *SON* (Figure 6a), whereas the outlier loci only resolved two groups, corresponding to the southern Baja peninsula combined with *SON* and the northern Baja peninsula combined with *SJ* (Figure 6b). Phylogenetic trees constructed from the same number of randomly selected neutral loci as there are outliers also recovered four populations (Figure S5), suggesting that the contrasting topologies recovered by the outlier and neutral loci are unlikely to be caused by differences in resolving power. By comparison, the main difference between the neutral and outlier trees for *Brahea* was that the former resolved individual sierras (Figure S6a), whereas the latter did not (Figure S6b). This appears to be related to genetic resolution as phylogenetic trees based on the same number of randomly selected neutral loci as outliers also failed to clearly resolve the sierras (Figure S7).

## 4 DISCUSSION

Comparative studies can provide valuable insights into processes that shape population genetic structure and thereby help to improve our understanding of how organisms may respond to ongoing environmental change. We therefore used GBS both to resolve taxonomic uncertainties and to characterize patterns of population structure in two closely related North American palm genera, *Washingtonia* and *Brahea*, on the Baja California peninsula, adjacent Mexican mainland and Guadalupe Island. Bayesian phylogenetic analysis supported the classification of *B. edulis* as a distinct species, but this was not the case for *W. filifera*–*W. robusta* and *B. armata*–*B. brandegeei*. Furthermore, population genetic analyses clustered the peninsular *Washingtonia* palms into two populations corresponding to the northern and southern peninsula, whereas in *Brahea* every sierra could be genetically distinguished. We also detected a relatively strong influence of ecologically mediated divergence in *Washingtonia* palms, with outlier loci correlated to temperature resolving a markedly different phylogenetic tree to neutral loci. Finally, we found evidence for two unidirectional long‐distance migration events in *Washingtonia*, in line with the previous suggestion that human‐mediated dispersal could have been disproportionately important in this genus (McClenaghan & Beauchamp, 1986; Minnich et al., 2011). None of these patterns could previously be detected using either allozymes (McClenaghan & Beauchamp, 1986) or classical plastid and nuclear markers (Klimova et al., 2017), suggesting that GBS and related approaches represent powerful tools for uncovering ecologically relevant population subdivision.

### 4.1 Phylogenetic relationships

The first aim of our study was to resolve phylogenetic relationships between and within *Washingtonia* and *Brahea* palms sampled from the Baja California peninsula and adjacent areas. As expected, the two genera were found to be deeply divergent, consistent with their having separated from one another at least 25–35 million years ago (Bacon et al., 2012; Baker & Couvreur, 2013. However, mixed support was found for currently recognized taxonomic relationships within each genus (Felger & Joyal, 1999; Henderson et al., 1995; Minnich et al., 2011). Starting with *Washingtonia*, Bayesian phylogenetic reconstruction uncovered two main lineages, the first corresponding to *W. robusta* from the north of the Baja peninsula together with *W. filifera*, and the second corresponding to *W. robusta* from the southern Baja peninsula and the Mexican mainland. Furthermore, the magnitude of divergence between *W. filifera* and *W. robusta* from the northern Baja peninsula was lower than that found between *W. robusta* from the northern Baja peninsula and the Mexican mainland (i.e., it was within the range found within a single species). Our data therefore lead us to the conclusion that *W. filifera* is more likely to represent the northernmost population of *W. robusta* than a separate species. This is in line with a previous study by Bacon et al. (2012), who found no differences between *W. filifera* and *W. robusta* at three plastid and three nuclear genes, and is also consistent with an apparent lack of reproductive isolation between these palms, as hybridization is common in cultivation (Hodel, 2014). Furthermore, a highly detailed morphological study of *Washingtonia* palms from 17 sites on the peninsula recently found no clear support for two distinct species based on 11 morphological characteristics but rather suggested the presence of a latitudinal morphological cline (Villanueva‐Almanza & Ezcurra, 2017).

Bayesian phylogenetic reconstruction of *Brahea* provided evidence in support of the species status of Guadalupe Island palms (*B. edulis*) although this was to some extent expected given the geological origin and geographic isolation of Guadalupe Island (Aleixandre, Hernandez‐Montoya, & Mila, 2013; Karhu, Vogl, Moran, Bell, & Savolainen, 2006; Klimova et al., 2017). Nonetheless, our data did not support the recognition of two separate species, *B. armata* in the northern peninsula and *B. brandegeei* in the southern peninsula. Again, this is consistent with a previous genetic study based on chloroplast and nuclear sequences, which also failed to separate the peninsular *Brahea* into two monophyletic groups (Klimova et al., 2017). One reason for this could be that the original taxonomy was based on relatively subtle morphological differences such as leaf coloration and inflorescence architecture (Felger & Joyal, 1999; Henderson et al., 1995) and these traits could potentially show plastic variation among populations in response to the prevailing environmental conditions (Roncal, Henderson, Borchsenius, Cardoso, & Balslev, 2012). One way to test this hypothesis would be to use reciprocal transplant or common garden experiments.

### 4.2 Patterns of population genetic structure

*Washingtonia* and *Brahea* palms from the Baja California peninsula provide a unique opportunity to explore the contributions of multiple potential drivers of population structure within an unusually heterogenous natural setting. However, a recent study based on nuclear and chloroplast genes was unable to recover sufficient levels of polymorphism to provide insights at the population level (Klimova et al., 2017). Fortunately, GBS allowed us to genotype over 25,000 SNPs, which after highly stringent filtering to retain only polymorphic, unlinked loci with a small proportion of missing data, left us with a total of 1,462 and 2,050 genome‐wide distributed SNPs in *Washingtonia* and *Brahea* respectively. These data allowed us to uncover contrasting and in some cases unexpected patterns of genomewide differentiation, with *Washingtonia* populations showing a clear north–south split, whereas in *Brahea* each individual sierra could be resolved.

We found evidence for four main groups of *Washingtonia* palms. As might be expected given the degree of geographic isolation, palms from the Mexican mainland and *SJ* formed separate clusters, but we did not anticipate finding two distinct palm lineages among sierras of the Baja California peninsula that were roughly evenly spaced along a latitudinal cline. Many plant and animal species on the peninsula show a similar north–south divide that has been linked to the temporary formation of a mid‐peninsula seaway around a million years ago (Lindell et al., 2006; Riddle et al., 2000). However, this is not strictly consistent with our results, as palms from *SSP* show membership to the northern cluster but are located to the south of where the seaway is believed to have been located. The reasons for this are not entirely clear. It is possible but fairly unlikely that the true location of the seaway was actually further south than is currently believed. Alternatively, *Washingtonia* could have been locally extirpated at *SSP* and subsequently recolonized from *SFSL* after the closure of the seaway. This explanation is plausible both because ecological niche modeling has shown major shifts in the distribution of suitable habitat over the past 100,000 years and *SSP* is currently represented by a single isolated oasis situated on the margins of a large area of unsuitable habitat (see Figure 6 in Klimova et al., 2017).

Our results for *Brahea* are in many respects more readily explained (Klimova et al., 2017). SPLITSTREE partitioned the palms into nine genetic clusters, each corresponding to a different sierra, while sNMF detected three main groups whose frequencies followed a clear cline along the peninsula, consistent with a significant pattern of isolation by distance (Mantel's *r* = .609, *p *<* *.001). This probably reflects the fact that the seeds of *Brahea* palms are primarily dispersed by water pulses that wash them short distances along canyons (Wehncke & López‐Medellín, 2014; Wehncke et al., 2009). By contrast, *Washingtonia* palms have edible fruit that are eaten by birds and small mammals and which were apparently also used by indigenous people (Cornett, 2008; Luna, 2012), thereby facilitating the dispersal of intact seeds between adjacent oases.

Another factor that could have contributed toward differences between *Washingtonia* and *Brahea* is demographic history. In particular, historical bottlenecks can lead to strong genetic drift and thereby contribute toward both the pattern and strength of population genetic structure (Futuyma & Kirkpatrick, 2017). Unfortunately, however, GBS and related approaches are not well suited to demographic reconstruction as tests for bottlenecks and population expansion are extremely sensitive to MAF thresholds and other aspects of the bioinformatic pipeline(s) used (Shafer et al., 2017). Nevertheless, we have little reason to believe that the two genera experienced markedly different recent demographic histories, as ecological niche modeling has shown that both *Washingtonia* and *Brahea* would have been locally restricted to similar areas during the LGM (about 22,000 years ago) and subsequently re‐established themselves across most of the peninsula (Klimova et al., 2017).

### 4.3 Ecologically mediated divergence

There is growing interest and empirical support for the notion that strong divergent natural selection can drive genomic divergence, ultimately leading in some cases to reproductive isolation and speciation (Beheregaray, Cooke, Chao, & Landguth, 2015; Lexer et al., 2014; Sork et al., 2016). The palms of Baja California are interesting in this regard because they reside at the extreme northern distributional limits of the mostly tropical Arecaceae and are therefore subjected to unusually dry, cold and generally suboptimal conditions (Hampe & Jump, 2011; Woolbright, Whitham, Gehring, Allan, & Beiley, 2014). Under such conditions, local adaptation can be a particularly important force in shaping patterns of divergence across the genome (Pannell & Fields, 2014; Savolainen, Lascoux, & Merilä, 2013). Our results are consistent with this notion and suggest that local adaptation may have contributed toward the population structure of *Washingtonia* in particular.

We originally hypothesized that the influence of ecologically mediated selection should be strongest on *Washingtonia* palms due to their stricter ecological requirements and relatively restricted geographic distribution (Minnich et al., 2011). In line with this, we detected significant associations between environmental variables and genetic distance in both genera, but these were stronger in *Washingtonia*. We also found that genetic distance was correlated to the mean temperature of the coldest quarter in *Washingtonia*, whereas in *Brahea* genetic distance was associated with precipitation of the driest quarter. Taken at face value, this difference would imply that, even though many of the *Washingtonia* and *Brahea* palms were sampled from the same oases, natural selection has influenced the two genera in different ways.

To explore this further, we used two different approaches to test for loci showing unusual associations with environmental variables. In both cases, we attempted to minimize the occurrence of false positives, either by controlling for the underlying population structure by introducing latent factors (in LEA) or using multivariate logistic regression (in SAMβADA), which reduces the occurrence of spurious genotype by environment associations (Stucki et al., 2016). These approaches identified different sized and largely non‐overlapping subsets of loci, but this is consistent with previous studies and reflects differences in the underlying methodologies and assumptions (Benestan et al., 2016; Feng, Jiang, & Fan, 2016; Nadeau, Meirmans, Aitken, Ritland, & Isabel, 2016). To capture as many outliers as possible, we therefore pooled all of the loci flagged by at least one approach and classified the remaining loci as neutral. Constructing phylogenetic trees separately for these two classes of locus revealed a clear difference in *Washingtonia*, with the neutral loci resolving four groups but the outlier loci only two groups. This finding is reminiscent of similar studies that likewise resolved different trees based on neutral and outlier loci (Funk et al., 2016; Keller et al., 2013; Matala, Ackerman, Campbel, & Narum, 2014). Such a pattern could be considered a footprint of selection as *SJ* and the northern peninsular sierras have diverged at the genomic background, while stabilizing selection appears to have resulted in very similar genotypes at the outlier loci.

The equivalent results for *Brahea* were less clear cut. Within the Baja peninsula, the outlier loci failed to resolve the individual sierras. However, phylogenetic trees based on the same number of randomly selected neutral loci also grouped the sierras together, suggesting that genetic differences between the sierras may be too weak to be resolved by a relatively small subset of SNPs. Either this could reflect weaker selective pressures on *Brahea* palms or alternatively the association between genetic distance and precipitation of the driest quarter in *Brahea* could be a type I error. Further insights into this and related questions could be gained from detailed physiological studies aiming to establish more clearly how the two palm genera respond to climatic extremes.

### 4.4 Patterns of long‐distance dispersal

A further unexpected pattern was revealed by cluster analyses of the GBS data. Overall, the majority of individuals were confidently clustered to their respective geographic groups and levels of admixture between the identified populations were low. However, several palms sampled from the northern sierras had genotypes that were clearly consistent with ancestry in the southern sierras, a pattern that is strongly suggestive of recent long‐distance dispersal. To explore this further, we used TREEMIX to infer the most likely number of migration events in both palm species. The results for *Washingtonia* were clear cut, with strong support being found for two distinct south to north migration events spanning around 450 km. By contrast, although one migration event was inferred for *Brahea*, this was not supported by three‐population tests and should therefore be treated as putative at best.

It is unlikely that these patterns could have resulted from differences in the natural dispersal abilities of the two palm genera because, even though *Brahea* is more dispersal limited, *Washingtonia* cannot be naturally dispersed over more than a few tens of kilometers. Furthermore, if natural agents such as birds or mammals were involved, one would not necessarily expect to find a bias in the direction of migration from north to south, which appears to be the case for the long‐distance migration events inferred in *Washingtonia*. However, as palms have a long history of relationship with humans and have been extensively used as a source of food, construction materials and more recently as ornaments in cities and gardens, several authors have speculated that humans may have been involved in spreading palms on the Baja peninsula (Cornett, 2008; Felger & Joyal, 1999; Levis et al., 2017; McClenaghan & Beauchamp, 1986; Minnich et al., 2011). Furthermore, the fruits of *Washingtonia* were extensively used as a food source by native people (Cornett 1987; Felger & Moser, 1985; Felger & Joyal, 1999), whereas *Brahea* fruit were less appreciated and have even been referred to as “useless” (Minnich et al., 2011), leading some authors to suggest that the indigenous people of the Baja peninsula may have dispersed *Washingtonia* but not *Brahea* prior to European contact (Cornett, 2008; Minnich et al., 2011). Our results are consistent with this hypothesis as long‐distance migration events were only inferred unequivocally in *Washingtoni*a. However, the two northern oases containing *Washingtonia* palms of southern ancestry are also both sites of colonial Spanish missions where agriculture and trade would have been especially well developed (Minnich et al., 2011). Consequently, it is not inconceivable that these genetic introductions could have occurred more recently, which is supported by the observation that the palms in question exhibited negligible admixture. Although there are clear precedents for human‐mediated dispersal of palms (Aschmann, 1957; Kondo et al., 2012; Rivera et al., 2013), we prefer not to speculate further at this point without additional archeological evidence or more detailed genetic data that would allow us to reliably date these migration events.

### 4.5 Limitations of the study

The recent development of cost‐effective methods for obtaining high‐quality genome‐scale data has stimulated growing interest in the genomic basis of ecological divergence. By greatly increasing genotyping coverage, approaches like GBS have made it possible to identify genomic regions and in some cases specific loci responsible for adaptive differences among populations (Savolainen et al., 2013). Nonetheless, a number of caveats need to be taken into account. For example, population structure, demographic history and the quality of the environmental data and biases caused by the genetic markers themselves can all lead to false‐positive results in outlier scans (Hoban et al., 2016).

First of all, disentangling IBE from neutral patterns of genetic variation can be challenging (Wang & Bradburd, 2014) because IBD can produce patterns similar to IBE when geography is correlated with environmental variation (Meirmans, 2012; Nadeau et al., 2016). We attempted to reduce this potential source of bias by analyzing only ecological variables that were significantly associated with genetic distance after having controlled for geographic distance. We further minimized the occurrence of false positives by controlling for population structure and demographic history by introducing latent factors into the LEA analysis and by implementing a multivariate logistic regression approach in SAMβADA.

Second, the accurate detection of locally adapted loci through genotype–environment associations also depends on accurate measures of different aspects of the ecological landscape. Low‐resolution environmental data may reduce the accuracy of results even if the selective environment is known (Hoban et al., 2016), so the resolution of the environmental data must be fine enough to adequately characterize each sampling locality. For this reason, we cross‐referenced fine resolution (~1 km scale) environmental data with GPS coordinates collected by ourselves at each oasis.

Third, in common with virtually all genotyping approaches including restriction enzyme‐based methods, GBS suffers from a number of potential sources of genotyping error (Andrews et al., 2016; Hoban et al., 2016). One of the most important of these is the presence of null alleles. These occur when a polymorphism within the restriction enzyme recognition site results in failure to cut the genomic DNA at that location. Alleles lacking the complete recognition site are not sequenced, which results in individuals who are heterozygous for the null allele appearing as homozygotes. Null alleles can downwardly bias estimates of genetic diversity, overestimate *F*_st_, and result in an increase in false positives in *F*_st_ outlier tests (Andrews et al., 2016). To overcome this limitation, we used two approaches that detect associations between genetic and environmental distances without estimating *F*_st_ (Frichot & François, 2015; Stucki et al., 2016).

A related issue is that stochastic processes during PCR can cause one allele to amplify more readily than the other at a given locus. This can lead to downstream genotyping errors, as heterozygotes can appear as homozygotes, or alleles containing PCR errors can be interpreted as true alleles. However, at least in theory, PCR should not systematically favor one allele over another at a given locus, and therefore, parameters estimated from a large number of loci are unlikely to be substantially biased (Andrews et al., 2016). Furthermore, although reduced genome representation approaches such as GBS provide far greater genomic coverage than their predecessors such as microsatellites or amplified fragment length polymorphisms, it is nevertheless important to bear in mind that only a fraction of the genome can be screened, which may result in some important targets of selection being missed (Narum, Buerkle, Davey, Miller, & Hohenlohe, 2013). However, we were more interested in broad patterns rather than in the nature of the specific loci under selection, and the contrasting topographies of neutral versus selected loci in *Washingtonia* suggest that at least for this genus our study was successful at detecting genomic regions influenced by selection. Classical approaches like common garden or reciprocal transplant experiments would provide a suitable basis for future confirmatory studies (Anderson et al., 2010; Savolainen et al., 2013).

## 5 CONCLUSIONS

Comparative studies can shed light on species‐specific properties that may influence dispersal and ecological divergence, while GBS offers an unprecedentedly detailed window on genome‐wide patterns of differentiation. By combining these approaches in iconic North American palms, we were able to uncover markedly different patterns of population structure in *Washingtonia* and *Brahea*, reveal associations between genetic distance and climatic variables, identify subsets of loci that appear to be under divergent ecologically mediated selection, and identify long‐distance migration events consistent with human‐mediated dispersal. None of these patterns could be detected with classical molecular markers, indicating the promise of approaches like GBS to dissect apart the contributions of different processes toward genome‐wide patterns of divergence.

## CONFLICT OF INTEREST

None declared.

## AUTHOR CONTRIBUTIONS

AK, AOR, and JIH designed the research; AK collected samples; AK performed molecular laboratory work; AK, DLJV, and JIH analyzed the molecular data; AK and JIH wrote the manuscript; AOR provided funding; all authors approved of the final version of the manuscript.

## DATA ACCESSIBILITY

All of the genotypes together with environmental data for the sampling locations are available from the Dryad Digital Repository: https://doi.org/10.5061/dryad.5vk6219.
