# Supplementary material for: Genotyping by sequencing reveals contrasting patterns of population structure, ecologically mediated divergence, and long‐distance dispersal in North American palms
Source: Ecol Evol. 2018 May 8;8(11):5873–90. doi: 10.1002/ece3.4125 (PMC6010798; doi:10.1002/ece3.4125)

Supplementary information for "Genotyping by sequencing reveals contrasting patterns of population structure, ecologically mediated divergence and long-distance dispersal in North American palms" by Klimova A, Ortega-Rubio A, Vendrami DLJ, Hoffman JI

Supplementary Table 1. Sampled *Washingtonia* and *Brahea* species and populations, with geo-references and the number of individuals collected from each location. Species designations are based on Minnich *et al.* (2011).

Species	Sierra	Sampling site	n samples	Latitude	Longitude
Washingtonia robusta	Sierra La Laguna (*SLL*)	SLL1	4	23.65115	-109.92985
		SLL2	3	23.7358694	-109.8433778
		SLL3	3	23.2783056	-110.0375556
		SLL4	2	24.0813917	-110.1125667
		SLL5	3	24.2391583	-110.2031806
	Sierra Mechudo (*SM*)	SM1	3	24.3339861	-110.9142389
		SM2	3	24.4493972	-110.7975083
		SM3	3	24.47165	-111.0027889
		SM4	3	24.4929972	-110.8073778
		SM5	3	24.4941611	-110.8123583
		SM6	2	24.7703194	-110.7470833
		SM7	3	24.8121389	-110.8141306
		SM8	2	24.8485222	-111.0791306
		SM9	3	24.9908611	-110.964311
	Sierra Giganta (*SG*)	SG1	2	25.7176722	-111.4340167
		SG2	3	25.755778	-111.516958
		SG3	3	25.935722	-111.409793
		SG4	4	25.97617	-111.466954
		SG5	2	26.0169417	-111.8621472
	Sierra San Pedro (*SSP*)	SSP1	4	26.9219139	-112.4075167
	Mainland (*SON*)	SON1	4	28.0170417	-111.0553167
		SON2	5	28.0069472	-111.1305389
	Sierra San Francisco+Sierra Libertad (*SFSL*)	SFSL1	4	28.0495	-113.084225
		SFSL2	3	28.7401417	-113.7531278
	Catavina (*CAT*)	CAT1	3	29.743054	-114.673552
		CAT2	2	29.749688	-114.742954
Washingtonia filifera	Sierra Juarez (*SJ*)	SJ1	4	32.1535667	-115.7888833
		SJ2	2	32.2634111	-115.8383611
		SJ3	4	32.3515944	-115.8281667
Washingtonia total	-	-	89	-	-
Brahea edulis	Guadalupe Island (*GI*)	GI1	8	28.9326278	-118.2837389
		GI2	4	29.0056861	-118.2495361
		GI3	10	29.1612556	-118.3213333
Brahea brandegeei	Sierra La Laguna (*SLL*)	SLL1	2	23.2218722	-109.8695028
		SLL2	3	23.228125	-109.8889611
		SLL3	3	23.2381111	-109.9518333
		SLL4	2	23.65115	-109.92985
		SLL5	4	23.6855528	-109.9450639
		SLL6	4	24.0813917	-110.1125667
		SLL7	3	24.2391583	-110.2031806
	Sierra Mechudo (*SM*)	SM1	2	24.47165	-111.0027889
		SM2	4	24.7703194	-110.7470833
		SM3	3	24.8121389	-110.8141306
	Sierra San Pedro (*SSP*)	SSP1	4	26.88801	-112.43786

	San Fransisco de la Sierra (*SSF*)	SFS1<br>SFS2	7<br>4	27.5942083<br>28.0495	-113.0333444<br>-113.084225
Brahea armata	Sierra Libertad (*SL*)	SL1<br>SL2<br>SL3	4<br>1<br>4	28.3109583<br>28.354546<br>28.7401417	-113.5291361<br>-113.489647<br>-113.7531278
	Sierra Asamblea (*SA*)	SA1	4	29.2616472	-114.0187889
	Catavina (*CAT*)	CAT1<br>CAT2	5<br>3	29.743054<br>29.749688	-114.673552<br>-114.742954
	Sierra San Pedro Martir (*SSPM*)	SSPM1<br>SSPM2<br>SSPM3<br>SSPM4	2<br>2<br>2<br>2	30.2765917<br>30.3169639<br>30.3240333<br>30.4983778	-115.2237278<br>-115.2629694<br>-115.2055861<br>-115.7677306
	Sierra Juarez (*SJ*)	SJ1<br>SJ2	4<br>1	32.1535667<br>32.2634111	-115.7888833<br>-115.8383611
Brahea total	-	-	101	-	-

Supplementary Table 2. Estimates of genetic differentiation among *Washingtonia* sierras. Pairwise F_{st} and associated P-values after table-wide FDR correction are shown above and below the diagonal respectively (* $P < 0.05$; ** $P < 0.01$; *** $P < 0.001$).

Sierra	*SM*	*SG*	*SJ*	*SSP*	*CAT*	*SLL*	*SFSL*	*SON*
SM	-	***	***	***	***	*	***	***
SG	0.07	-	***	**	**	**	**	***
SJ	0.68	0.70	-	**	**	***	***	***
SSP	0.57	0.57	0.62	-	ns	***	ns	**
CAT	0.32	0.28	0.48	0.11	-	***	ns	***
SLL	0.02	0.04	0.68	0.54	0.28	-	**	***
SFSL	0.38	0.36	0.46	0.05	-0.01	0.35	-	***
SON	0.58	0.61	0.86	0.73	0.55	0.58	0.55	-

Supplementary Table 3. Estimates of genetic differentiation among *Brahea* sierras. Pairwise F_{st} and associated *P*-values after table-wide FDR correction are shown above and below the diagonal respectively (* $P < 0.05$; ** $P < 0.01$; *** $P < 0.001$).

Sierra	*SSP*	*CAT*	*SM*	*SLL*	*SJ*	*SL*	*SSF*	*SA*	*SSPM*
SSP	-	**	**	***	**	**	**	*	**
CAT	0.14	-	***	***	**	***	***	*	***
SM	0.12	0.12	-	***	***	***	***	***	***
SLL	0.13	0.17	0.08	-	***	***	***	***	***
SJ	0.24	0.15	0.23	0.27	-	**	***	**	**
SL	0.12	0.08	0.12	0.16	0.17	-	***	**	***
SSF	0.08	0.07	0.08	0.12	0.16	0.04	-	***	***
SA	0.16	0.06	0.14	0.19	0.16	0.06	0.07	-	**
SSPM	0.22	0.13	0.21	0.25	0.21	0.15	0.15	0.12	-

Supplementary Table 4. *R*esults of three-population admixture tests for *Washingtonia*. Only Z scores below -1.96, $P < 0.05$ are shown.

Target	Source1	Source2	f3	std.err	Z score
SFSL	SSP	SG	-0.0262	0.0017	-15.5763
SFSL	SSP	SLL	-0.0254	0.0019	-13.4876
SFSL	SSP	SM	-0.0259	0.0019	-13.3539
SFSL	SSP	SON	-0.0110	0.0023	-4.8111
SFSL	SJ	SM	-0.0099	0.0030	-3.2921
SFSL	SLL	SJ	-0.0096	0.0029	-3.2679
SFSL	SJ	SG	-0.0099	0.0031	-3.2416
CAT	SSP	SG	-0.0336	0.0022	-15.0415
CAT	SSP	SLL	-0.0313	0.0022	-13.9963
CAT	SSP	SM	-0.0305	0.0023	-13.3949
CAT	SJ	SG	-0.0229	0.0024	-9.5212
CAT	SLL	SJ	-0.0211	0.0024	-8.6932
CAT	SJ	SM	-0.0202	0.0025	-8.1145
CAT	SFSL	SG	-0.0091	0.0014	-6.3236
CAT	SFSL	SLL	-0.0077	0.0013	-5.8017
CAT	SFSL	SM	-0.0064	0.0013	-4.8042
CAT	SSP	SON	-0.0126	0.0030	-4.2398

Supplementary Figure 1. The distribution of the number of SNPs in the final dataset mapping to each scaffold in the *P. dactylifera* reference genome.

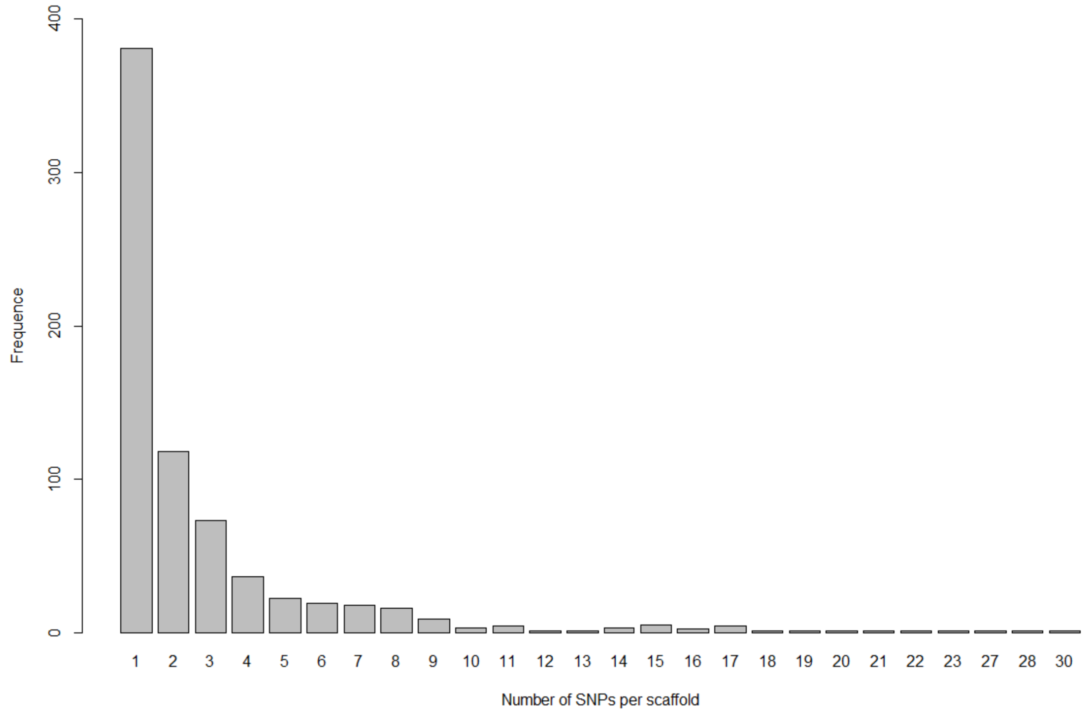

Supplementary Figure 2. Number of polymorphic SNPs shared for *Washingtonia* and *Brahea* palms (Note: 514 diagnostic SNPs are excluded from the figure).

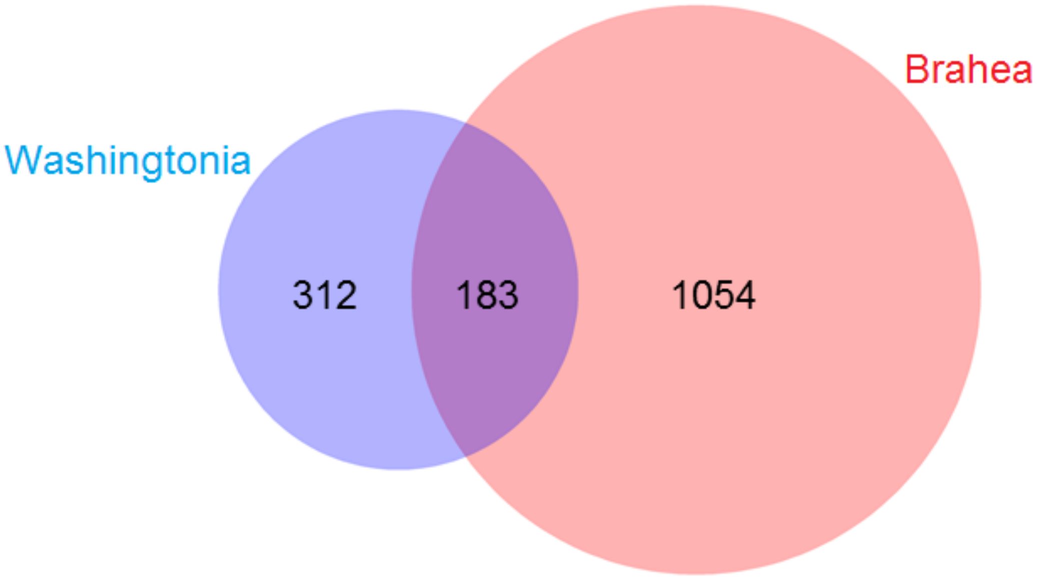

Supplementary Figure 3. Population structure as inferred with sNMF of (a) *Washingtonia* and (b) peninsular *Brahea* palms. Abbreviations correspond to sierras names as presented in Table S1 and Figure 1.

(a)

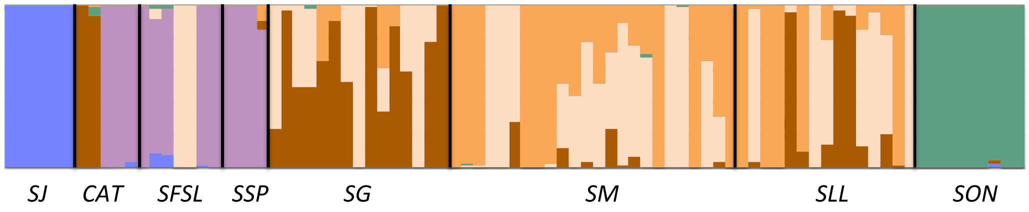

(b)

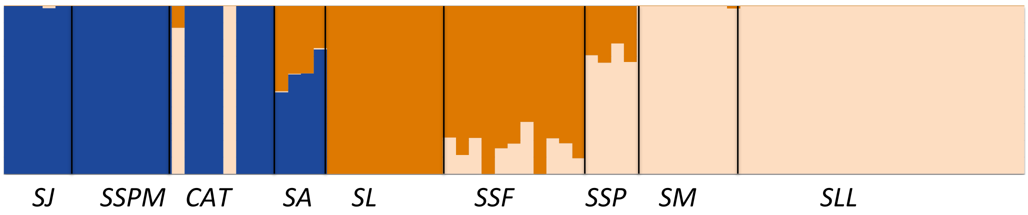

Supplementary Figure 4. Increase in log-likelihood as a function of the number of migration events between one and ten estimated in TREEMIX for *Washingtonia* in grey and for *Brahea* in black. Asterisks indicate the point after which successive stepwise comparisons of log likelihood values became insignificant (likelihood-ratio tests, $P > 0.05$).

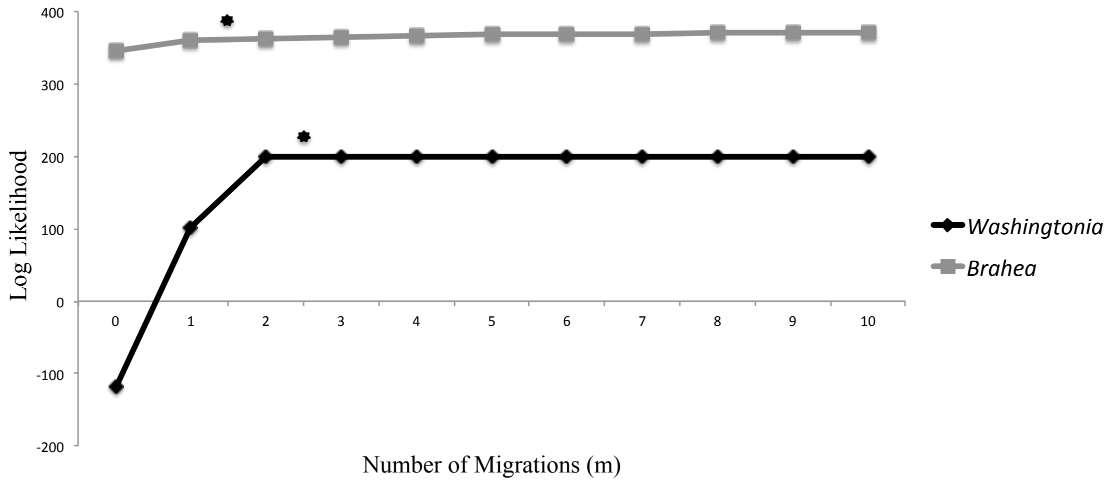

Supplementary Figure 5. Phylogenetic trees for *Washingtonia* based on three different subsets of 97 randomly selected neutral loci.

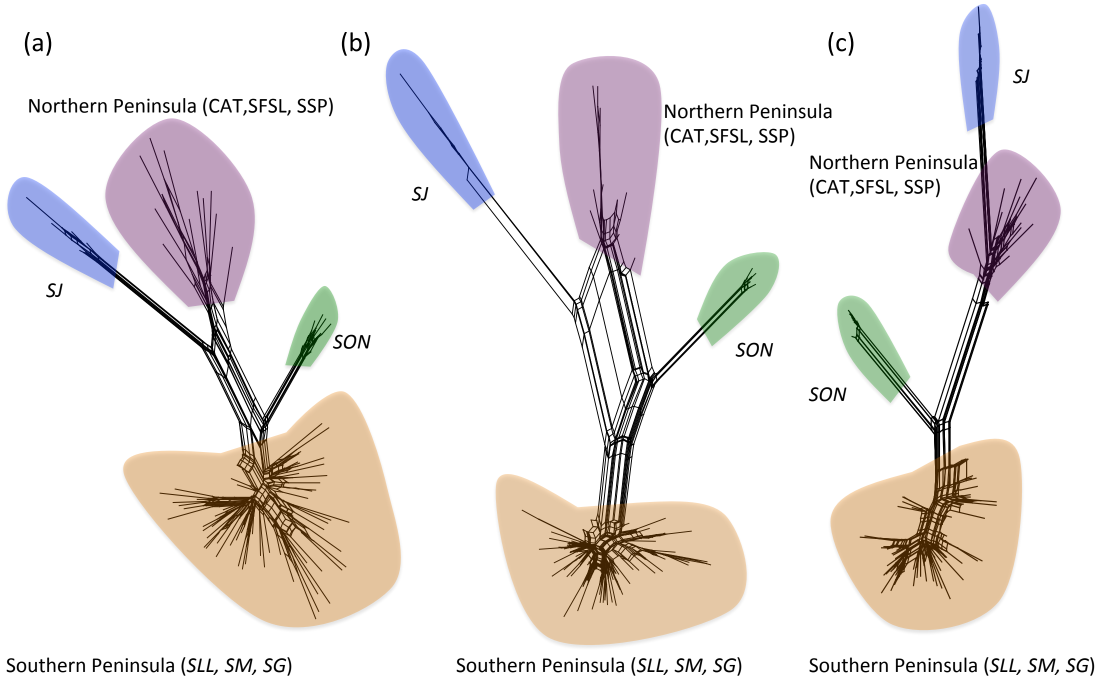

Supplementary Figure 6. Phylogenetic networks constructed separately for *Brahea* using (a) neutral loci; (b) outlier loci.

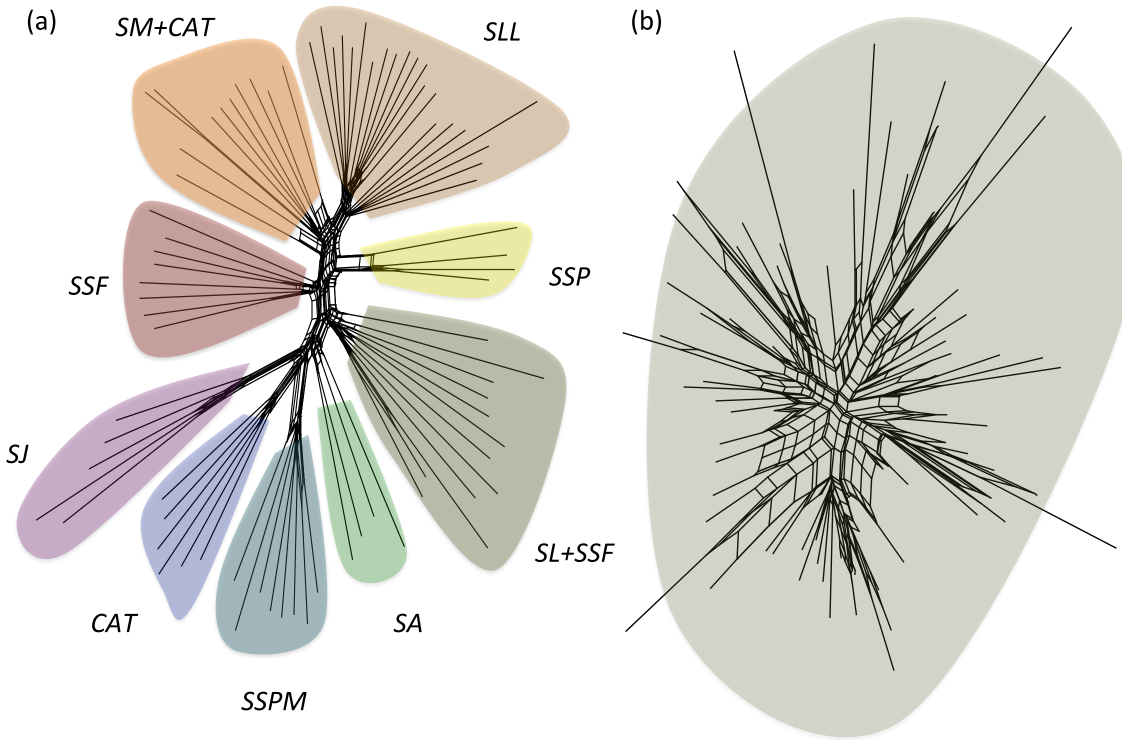

Supplementary Figure 7. Phylogenetic trees for *Brahea* based on three different subsets of 51 randomly selected neutral loci.

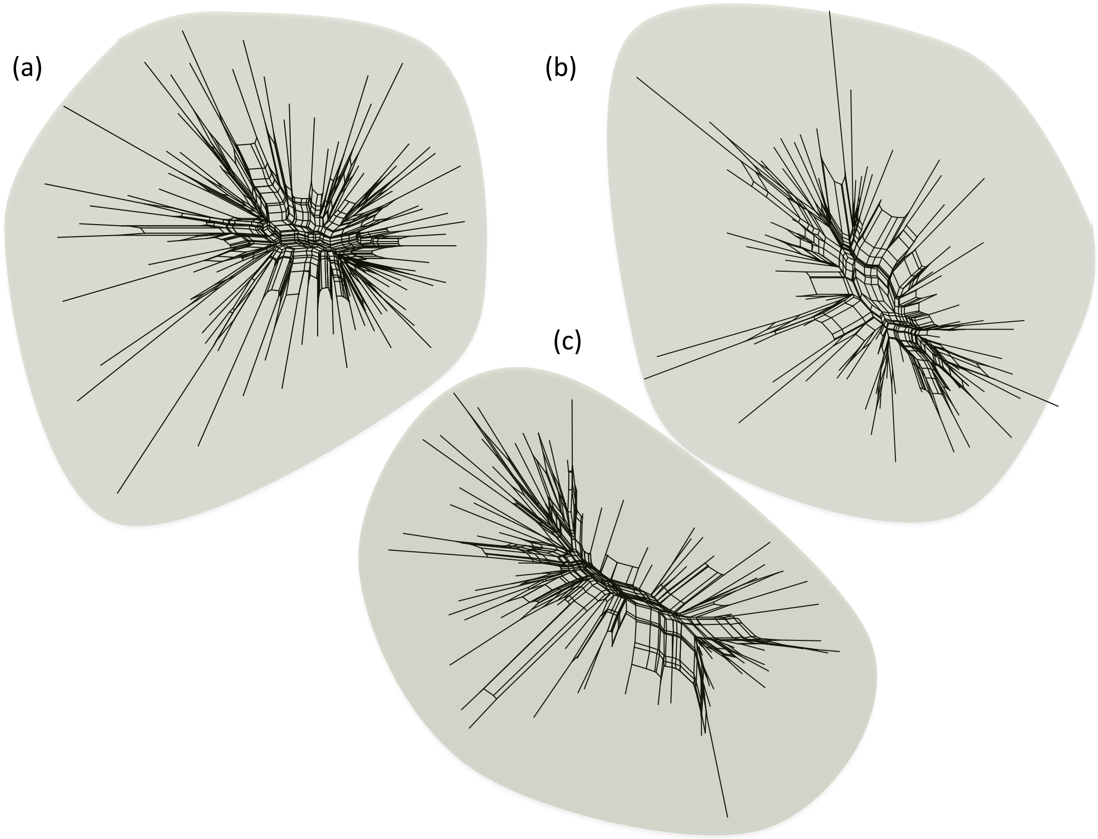

Supplement: Supplementary file 1 [file ECE3-8-5873-s001.pdf]
